# Facile synthesis of antibiotic-functionalized gold nanoparticles for colorimetric bacterial detection†

**DOI:** 10.1039/d1ra01316e

**Published:** 2021-04-15

**Authors:** Charlotte N. Elliott, María Cecilia Becerra, J. Craig Bennett, Lori Graham, M. Jazmin Silvero C., Geniece L. Hallett-Tapley

**Affiliations:** Department of Chemistry, St. Francis Xavier University P.O. Box 5000, Antigonish Nova Scotia Canada ghallett@stfx.ca; Departamento de Ciencias Farmacéuticas, Facultad de Ciencias Químicas, Universidad Nacional de Córdoba Córdoba X5000 Argentina jazmincompagnucci@gmail.com; Instituto Multidisciplinario de Biología Vegetal, IMBIV, CONICET Argentina; Department of Physics, Acadia University P.O. Box 49, Wolfville Nova Scotia Canada; Department of Biology, St. Francis Xavier University P.O. Box 5000, Antigonish Nova Scotia Canada

## Abstract

The development of quick and efficient methods for the detection of pathogenic bacteria is urgently needed for the diagnosis of infectious diseases and the control of microbiological contamination in global waterways, potable water sources and the food industry. This contribution will describe the synthesis of gold nanoparticles and their conjugation to broad spectrum, polypeptide and β-lactam antibiotics that function as both reducing agents and surface protectants (ATB@AuNP). Nanoparticle colloids examined using transmission electron microscopy are generally spherical in shape and range from 2–50 nm in size. Dynamic light scattering and infrared spectroscopy were also used to confirm encapsulation of the AuNP surface by antibiotic molecules. ATB@AuNP were then used to detect 3 common pathogenic bacterial species: *Staphylococcus aureus, Pseudomonas aeruginosa,* and *Escherichia coli*. The colour of the AuNP colloid was monitored visually and using UV-visible spectroscopy. A red shift of the UV visible absorbance and a visible colour change following introduction of each pathogen is indicative of ATB binding to the bacteria surface, ascribed to AuNP agglomeration. This work demonstrates that ATB@AuNP may be an efficient and high throughput tool for the rapid detection of common bacterial contaminants.

## Abstract 2

The following contribution will discuss a novel and rapid synthetic route for the generation of antibiotic functionalized gold nanoparticles and examine the application of these materials as colorimetric sensors for bacterial pathogens.

## Graphical abstract

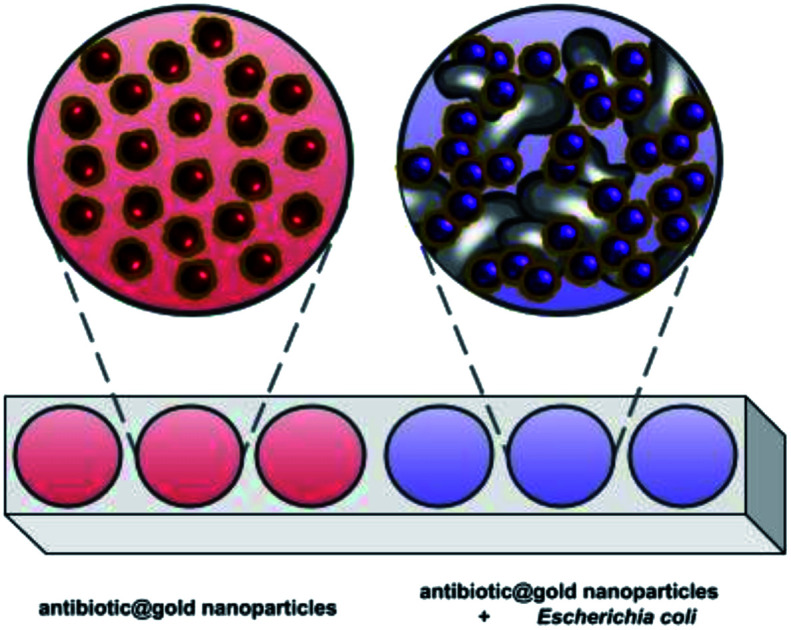

## Introduction

The Global Antimicrobial Surveillance System (GLASS) has estimated that in 2020 approximately half a million individuals were afflicted by antibiotic resistant bacterial infections.^1^ Unfortunately, higher infection mortalities are a direct consequence of the emergence of resistant mechanisms and rapid transfer between bacterial strains. Thus, there is an urgent need to develop a sensitive detection method that provides prompt results. Ideally, these procedures are capable of revealing bacterial contamination in food and water sources to mitigate infection, or in biological fluids to enable the rapid implementation of treatment protocols. Ideally, bacterial presence would be discovered prior to food commercialization, water consumption or worsening health of infected patients (*e.g.*, sepsis complications).

The benefits of developing a rapid, and sensitive, bacterial detection system are numerous. Traditional detection routes in clinical laboratory settings and microbiological food analysis are disadvantageous from both a time and cost perspective. Many of these techniques require lengthy incubation times (most >18 h), preparation of culture media and the cooperative use of microscopy.^2,3^ During this time, the potential exists for increased bacterial load. MALDI-TOF mass spectrometry applied for analysis of the proteomics of bacterial strains has enabled reduced detection times. However mass spectrometry is comprised of costly infrastructure and requires samples to be transported to the instrumentation site for analysis.^4^ On the other hand, the fabrication of portable technologies^5,6^ and PCR-based tests^7^ is ongoing, but both remain in the prototype stage of development due to high expenditures associated with these methods.

Recent works report that infection biomarkers, such as antibodies, can be efficiently detected by nanometric structures,^8–11^ such as gold, silver, or magnetic nanoparticles. Molecules with associated bacterial selectivity can be conjugated to the surface of the nanospecies. This interaction affords a level of bacteria selectivity and allows for efficient isolation or detection *via* emission of a visible signal. Particularly, gold nanoparticles (AuNP) have garnered interest due to their unique optical properties, as well as facile synthesis and functionalization.^12^ Indeed, examples showcasing the versatility of AuNP in bacterial detection are abundant. Padmavathy *et al.* have demonstrated the use of nanometric arrays in the detection of *Escherichia coli* (*E. coli*), using rapidly synthesized (<1 h) AuNP bound to a 20-base oligonucleotide.^13^ Similar AuNP arrays have been designed to selectively detect dipicolinic acid, a unique biomarker of bacterial spores.^14^ Other AuNP prototypes rely on conjugation to antibodies to enable specific detection of *Staphylococcus aureus* (*S. aureus*) using surface-assisted laser desorption/ionization mass spectrometry (SALDI-MS).^15^ The latter contribution exemplifies the advantage of AuNP nanosensors and the flexible nature of this approach. Enzymatic functionalization has been used to improve the detection properties of bacterial nanosensors, identifying low levels of both *S. aureus* and *E. coli* in potable water sources (minimum 1000 CFU mL^−1^).^16^ More recent advances have demonstrated the promise of the enzymatic method in the design of reaction strips in an effort to further expedite the bacterial identification and improve on-site detection. However, these novel designs are not without drawbacks, including lengthy detection times (∼30 min) and decreased bacterial selectivity.^17^ As can be seen, several nano-based bacterial methods exist, but many require intensive and costly synthetic pathways or employ complex detection procedures.

In an effort to address many of the aforementioned obstacles encountered by current nanosensors, the following contribution will discuss a novel and optimized method for routine bacterial detection. The nanomaterials presented herein employ a straightforward and cost-effective pathway of synthesizing AuNP functionalized with small, commercially available antibiotic (ATB) molecules. The ATB-functionalized gold nanoparticles (ATB@AuNP) give rise to vibrantly coloured colloidal solutions that vary according to AuNP shape, AuNP size, ATB ligand and ATB concentration. Due to the binding selectively of the ATB to some bacterial strains (depending on their structure and the components of the bacterial membrane), a colorimetric detection strategy has been employed to function as an initial screening tool for the identification of common pathogenic bacteria in water and, at a later stage, food or biological fluids.

## Materials and methods

### Synthesis of ATB@AuNP

**Fig. 1:**
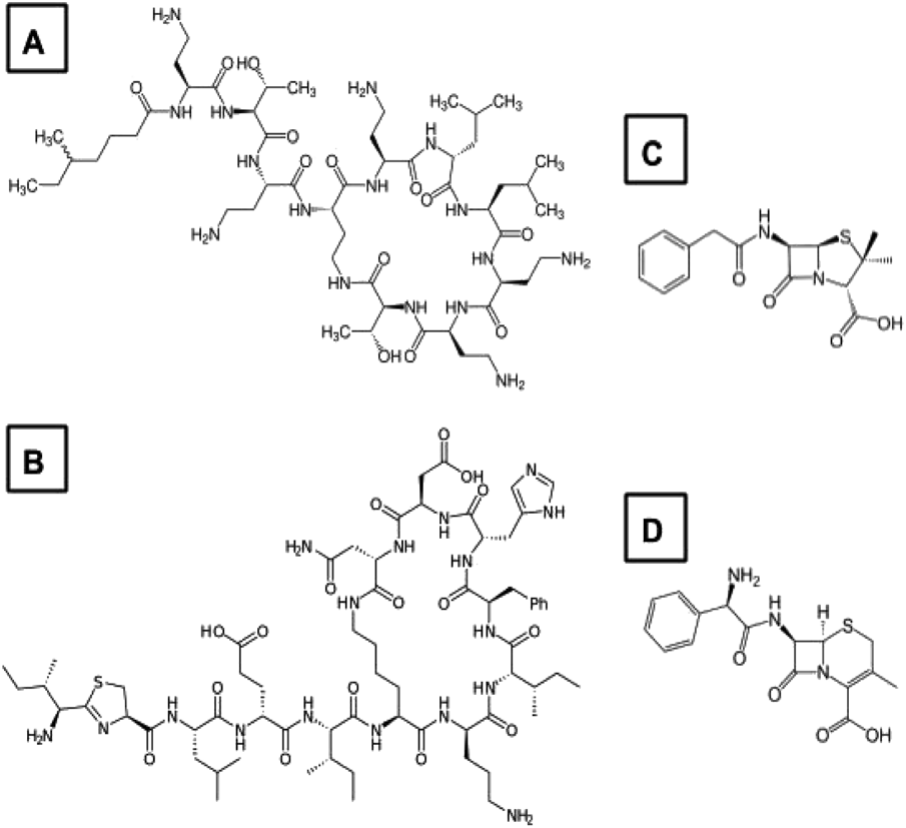
Chemical structures of the ATB used in the synthesis of ATB@AuNP: polymyxin (A), bacitracin (B), penicillin (C) and cephalexin (D).

Synthesis of AuNP were carried out through reduction of a 10 mM, aqueous HAuCl_4_·3H_2_O (MilliporeSigma) solution using 0.1 mM, aqueous ATB solutions as reducing agents and stabilizers. This one pot reaction was adapted from Silvero *et al.*^18^ Samples were prepared in 1.5 mL polypropylene microcentrifuge tubes using a heated water bath (Fig. S1†). Low cost, commercially available ATB with high amide group functionalization were selectively chosen to the known affinity of amides for the AuNP surface.^18,19^ Rani *et al.* implemented a similar approach to enable the surface modifications of AuNP by amino acid residues, specifically Arg and Glu, void of sulfur.^20^ The ATB implemented in this work are presented in Fig. 1. Polymyxin B (A; VWR Canada) and bacitracin (B; MillporeSigma) are classified as polypeptide ATB and exhibit selectivity for the phospholipids in bacterial membranes. Penicillin G sodium salt (C; MilliporeSigma) and Cephalexin (D; VWR Canada) are β-lactam ATB that bind to the penicillin-binding proteins in bacteria. Four AuNP hybrids were synthesized: poly@AuNP, baci@AuNP, peni@AuNP, and cepha@AuNP as a result of interaction with polymyxin, bacitracin, penicillin, and cephalexin, respectively. Fresh ATB solutions were prepared daily. HPLC grade water (Fisher Scientific Canada) was used in the preparation of all stock solutions and for spectroscopic analysis. ATB-specific experimental procedures are summarized in Table 1.

**Table 1:** Optimized experimental conditions for the synthesis of ATB@AuNP

Conditions	Penicillin	Cephalexin	Bacitracin	Polymyxin
Water bath (°C)	90	85	90	85
0.1 mM ATB (μL)	90	960	900	900
10 mM Au^3+^ (μL)	30	40	120	120
H_2_O (μL)	900	—	—	—
Reaction time (min)	2	2	15	15
Additional experimental details	Leave microcentrifuge tube open during heating			
			Sonicate mixture 30 min prior to heating	
				Add 4 μL of NaOH 1M after 8 min of heating
		
		
		

### Characterization of the ATB@AuNP

The formation of AuNP colloids was confirmed using a dual beam Cary 100 UV-visible spectrometer, using H_2_O as the reference sample. ATB conjugation was substantiated *via* infrared spectroscopy (Thermo Nicolet Nexus 670 FT-IR spectrometer equipped with Omnic Software). AuNP size was assessed using dynamic light scattering (Malvern Zetasizer Nano). TEM images, diffraction patterning and energy dispersive X-ray spectra (EDS) were collected on a Philips CM30 transmission electron microscope set at 250 kV acceleration voltage and equipped with Gatan Orius 832 CCD camera using Digital Micrograph 3. Average nanoparticle sizes were determined using ImageJ software^21^ and a minimum of 200 measurements. TEM samples were prepared by direct deposition of 10 μL of the ATB@AuNP colloids onto the carbon side of a carbon-coated copper TEM grid. Baci@AuNP required a 1 : 3 dilution (in water) ratio prior to TEM sample preparation. Centrifuged, washed and resuspended (in the same volume of water) ATB@AuNP samples were used for all characterization experiments.

### Bacterial detection

Using a sterile 96 well culture plate, 100 μL of each ATB@AuNP was added to 100 μL of 10^6^ CFU mL^−1^*S. aureus ATCC 29213*, *Pseudomonas aeruginosa* (*P. aeruginosa*) *191150* and *E. coli ATCC 25922*, respectively. 0.1 M HEPES buffer (pH 7.2) was used to prepare bacterial suspensions cultured overnight in tryptone soy broth and was also used as an experimental control. UV-visible spectroscopy was performed to assess colour changes induced by interaction of the microorganism and AuNP surface. The absorption spectrum of each ATB@AuNP was collected prior to and after 5 minutes of bacterial culture exposure. The experimental procedure was completed on two separate days, with assays completed in triplicate each day (*n* = 6).

## Results and discussion

### Synthesis of ATB@AuNP

Successful synthesis of all ATB@AuNP colloids requires constant heating, as well as interaction with atmospheric oxygen by keeping the reaction vessel open and exposed to air. Synthetic attempts using sealed (closed) microcentrifuge tubes were unsuccessful and corroborate the requirement for the presence of atmospheric oxygen for successful AuNP formation. ATB@AuNP comprised of polypeptide antibiotics with lipophilic tales (*e.g.*, poly@AuNP and baci@AuNP) require an additional sonication process, prior to heating, to facilitate nanoparticle formation. Previous studies have shown that bacitracin sonication is necessary to expose reducing groups on the ATB surface,^22^ enabling the formation of AuNP nucleation sites. This conclusion is strongly supported by the current findings.

The pH of the initial ATB solution, influential on the ATB surface charge, is also a critical parameter for spatial and temporal control of the AuNP colloids. Particularly, when bacitracin pH is below the known ATB isoelectric point (pH = 6.4), the exterior of the molecule will possess positive charges that are apt to bind with [AuCl_4_]^−^. This strong baci/AuCl_4_^−^ coordination facilitates formation and stability of the AuNP colloid.^22^ Likewise, an initial acidic polymyxin solution is required. At physiological pH, the polymyxin chemical structure consists of two hydrophobic domains (the N-terminal fatty acyl chain^23^ and the d-Phe-l-Leu^24^ segment) separated by segments of polar (Thr) and cationic (l-α-γ-diaminobutyric acid) residues. The polymyxin B molecule is folded such that the hydrophilic and hydrophobic domains form two distinct faces, thereby conferring the structural amphipathicity to enable nanoparticles formation.^25,26^ However, the final stage of polymyxin mediated synthesis (∼8 min) requires an increase to pH 10. This additional condition likely causes a negative charge on the amino residues to permit selective ATB binding to the AuNP surface.^27^

The described AuNP synthetic procedure affords a wide array of colloidal solutions ranging from light red to deep purple, dependent on ATB concentration (peni@AuNP is shown in Fig. S2†). Bacterial assays were performed using AuNP formed from the optimized HAuCl_4_ : ATB ratios that afforded the highest absorption and optimal stability (presented in Table 1).

### Characterization of ATB@AuNP

**Fig. 2:**
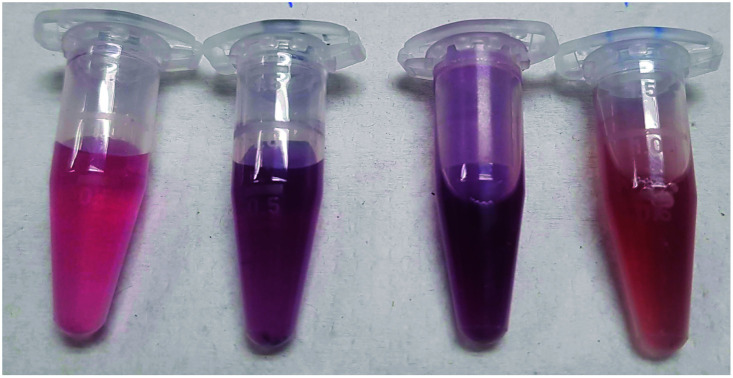
Image illustrating the vibrant, and visible, colour of ATB@AuNP synthesized using the conditions noted in Table 1. From left to right: peni@AuNP, cepha@AuNP, baci@AuNP and poly@AuNP.

Successful ATB@AuNP synthesis was initially confirmed using UV-visible spectroscopy with focus on the characteristic region of the surface plasmon band (SPB) (∼530–540 nm). The *λ*_max_ of the ATB@AuNP SPB are presented in Table 2. The nanoparticle colloids all imparted a characteristic red to red-violet colour due to the surface plasmon resonance properties of the material (Fig. 2); slight variations in the ATB@AuNP colour arise from differing electrostatic interaction between the ATB ligand and the AuNP surface.^18^ The SPB intensity and *λ*_max_ was also used to assess ATB@AuNP colloidal stability with all SPB absorption bands remaining unaffected over a monitoring period of 1 month. This spectroscopic data is indicative of high colloidal stability. The apparition of the SPB and its associated colour is of high importance as this absorption will be exploited for the colorimetric bacterial detection.^28^

**Table 2:** Characterization data for ATB@AuNP

Experimental technique	peni@AuNP	cepha@AuNP	baci@AuNP	poly@AuNP
SPB (*λ*_max_; nm)	549	549	557	520
Abs max (UV-vis)	1.0	1.3	2.4	1.2
[AuNP] (nM)^a^	100	34	1.4 × 10^3^	124
TEM shape/size (nm)/% population	Spheres/5.0 (55%)	Spheres/3.2 (62%)	Spheres/2.7 (62%)	Spheres/4.7 (100%)
	Irregular/60.0 (45%)	Hexagon/49.9 (38%)	Hexagon/36.3 (26%)	
		Triangle/68.5 (5%)	Triangle/21.9 (12%)	
DLS ave. Sizes (nm)/% population	108 (98.6%)	64 (94%)	406 (98%)	76 (100%)
	4483 (1.4%)	8 (6%)	5222 (2%)	

a AuNP concentration was approximated using initial [Au^3+^] and TEM data. See Fig. S13 for details.

Confirming ATB functionalization of the AuNP surface is also critical towards design of this novel colorimetric sensor. Surface modification was examined using infrared (IR) spectroscopy on centrifuged, washed and resuspended (in the same volume of water) ATB@AuNP solutions. IR spectra afforded a signal at 1760 cm^−1^ due to the presence of the β-lactam ring of penicillin or cephalexin, respectively. However, the characteristic 1690 cm^−1^ C=O stretching for cephalexin and penicillin (attributed to the amide functionality of its structure) is no longer observed (Fig. S3†). Similar spectroscopic observations have been reported by Manelli *et al.* and were considered acceptable confirmation of cephalexin conjugation to the nanoparticle surface.^29^ Reported ^1^H NMR analysis of cepha@AuNP has shown that these colloids are comprised of an oxidized ATB form.^30^ This data supports the hypothesis that the oxophilic nature of β-lactam antibiotics is responsible for Au^3+^ reduction, with preferential AuNP binding to the amide group of penicillin and cephalexin, respectively.

IR frequencies corresponding to the ATB were also observed for poly@AuNP and baci@AuNP. The IR data are in agreement with previously reported values.^31,32^ Poly@AuNP afforded signals corresponding to C–H stretching (2930 cm^−1^), as well as the amide C=O groups (amide I – 1646 cm^−1^, amide II – 1525 cm^−1^, amide III – 1243 cm^−1^).^33–35^ Baci@AuNP afforded similar C–H and amide C=O stretching vibrations, as well as additional signals assigned to the C=C groups (1540 cm^−1^), aromatic C–C stretches (1660 cm^−1^) and C–O vibrations (1100 cm^−1^).

**Fig. 3:**
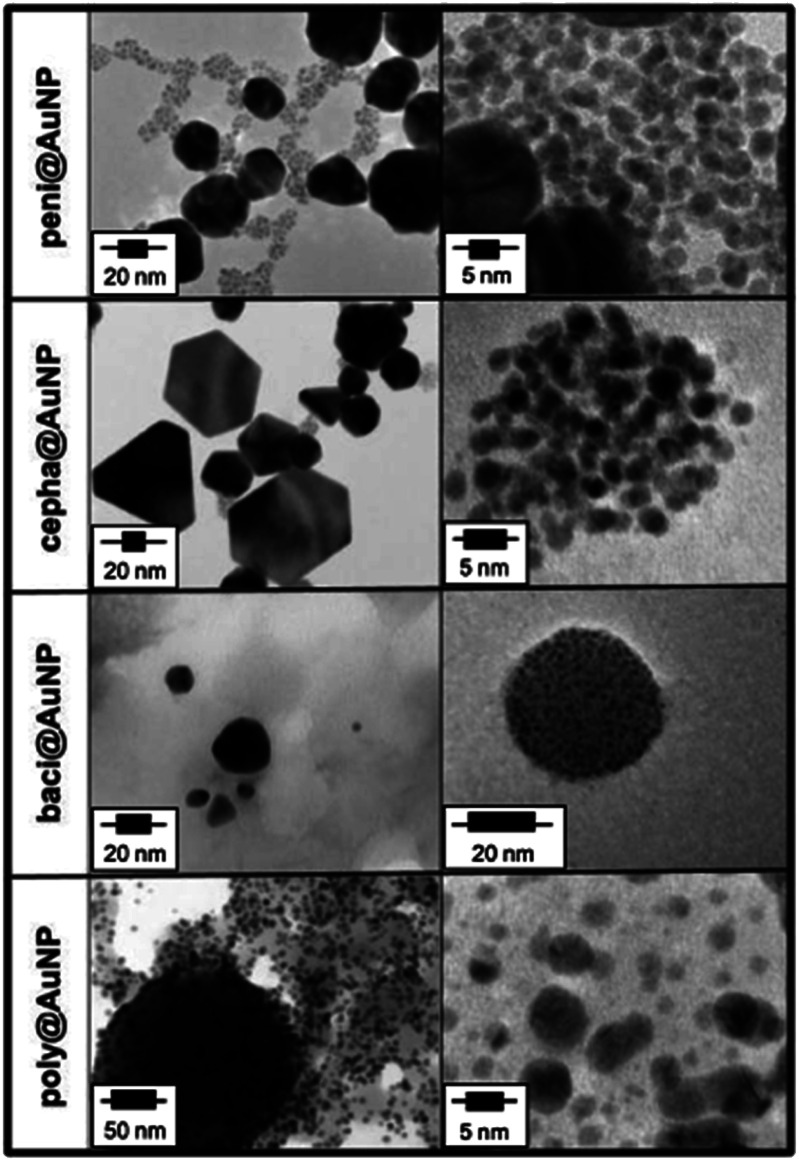
Representative TEM for ATB@AuNP.

TEM and dynamic light scattering (DLS) were used to affirm both the size and shape of the AuNP. Representative TEM pictures of ATB@AuNP hybrids are presented in Fig. 3 and corresponding size and morphology data in Table 2. Each ATB@AuNP colloid presented unique (and reproducible) size and shape characteristics. DLS analysis of peni@AuNP reveals the presence of two size populations, corroborated by TEM. The first group is comprised of a limited number of Au nanostructures that are irregular and polydisperse in size (60.0 ± 8.5 nm). Conversely, the second group is mainly constituted of densely populated, monodisperse, spherical AuNP (5.0 ± 0.2 nm) engulfed in an amorphous matrix (Fig. 3), attributed to penicillin functionalization of the Au surface.

DLS analysis of cepha@AuNP revealed three, distinguishable size populations. Closer examination using TEM revealed a higher fraction of these particles assume a triangular (68.5 ± 6.4 nm) or hexagonal (49.9 ± 6.1 nm) morphology, as compared to peni@AuNP. An abundant monodisperse, spherical population was also observed (3.2 ± 0.3 nm).

The TEM images obtained for polymyxin and bacitracin-functionalized AuNP were considerable different than those obtained for colloids constructed using β-lactam ATBs. TEM images obtained for baci@AuNP show a thin film of an amorphous, spherical structures (Fig. S4†). This unique morphological state can be attributed to nanoemulsion that forms following sonication of the bacitracin/Au^3+^ solution prior to heating.^36,37^ Small, crystalline particles (2.7 ± 0.7 nm) are embedded in the amorphous spheres, as well as larger hexagonal (36.3 ± 6.7 nm) and triangular (21.9 ± 5.0 nm) plates. Similar results were concluded for poly@AuNP, with the spheres encasing primarily smaller, more uniform, crystalline structures (4.7 ± 1.6 nm). Diffraction rings obtained for the encapsulated crystalline structures are consistent with the Au (111) surface.^38–40^ EDS analysis of composition of these materials do show some contributions from elemental Au, as expected. A representative EDS analysis for baci@AuNP is shown in Fig. S5.† HR-TEM of the ATB/AuNP samples (Fig. S6–S9†) also validate the metallic particles as Au nanospecies. In all cases, HR-TEM images illustrate lattice fringes confirming the crystalline structure of the metallic nanospecies. FFT and FFT filtered images of cepha@AuNP (Fig. S6†) reveal diffraction spots corresponding to the Au (111) surface with fringe spacing of 2.4 Å, consistent with the spacing of (111) planes for Au. Similarly, FFT analysis of both peni@ (Fig. S7†) and poly@AuNP (Fig. S8†) illustrate the crystalline nature of these particles, attributed with the Au (110) surface. HR-TEM of baci@AuNP (Fig. S9†) also presents lattice fringes indicative a highly crystalline metallic nanospecies, further corroborated by diffraction ring analysis as the Au (111) surface.

**Fig. 4:**
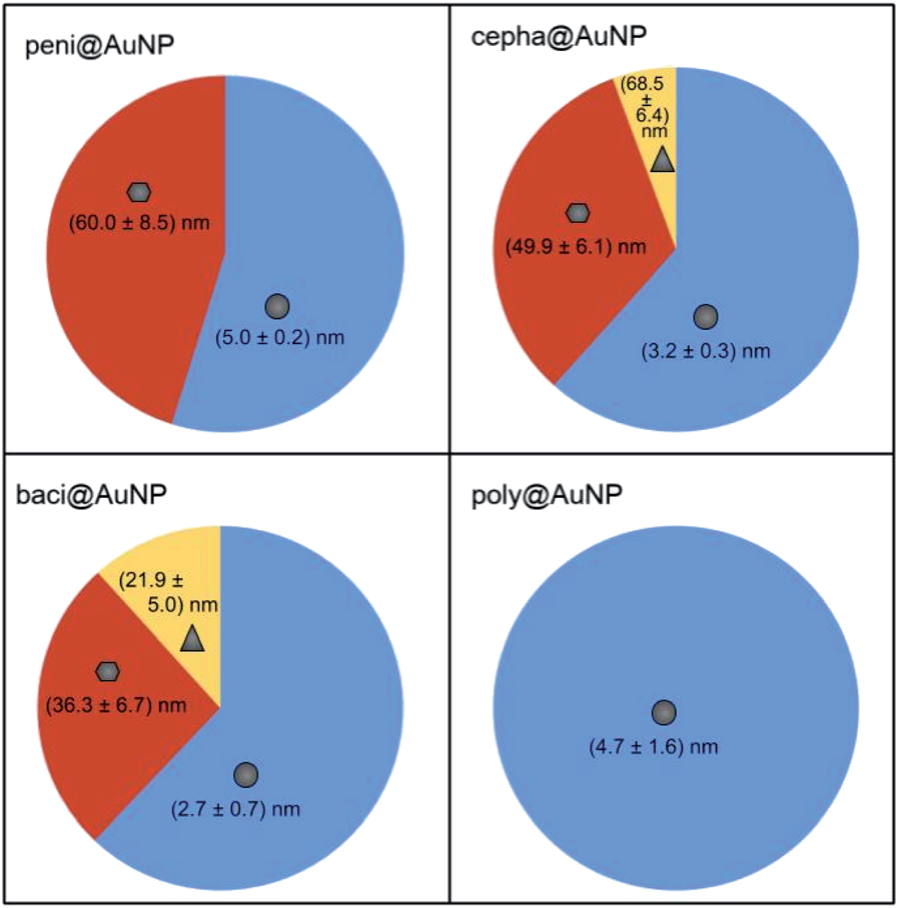
Schematic representation of ATB@AuNP size and morphology distribution.

DLS analysis of the bacitracin and polymyxin-protected colloids was unable to accurately illustrate the AuNP populations observed *via* TEM. Particle sizes of ∼406 nm for baci@AuNP and ∼70 nm for poly@AuNP are well outside the size range of nanostructures observed by TEM. As can be seen in Fig. S4,† the observed nanoemulsion for baci@AuNP is comprised of groups of 3–5 nanodroplets ∼100 nm in diameter. Thus, DLS is most likely identifying the additive diameter of such nanodroplet cohorts, affording inflated DLS size distribution values. A schematic representation of the calculated size distribution and morphologies of each ATB@AuNP is presented in Fig. 4.

### Assessment of colorimetric bacterial detection

The colorimetric sensor properties of the ATB@AuNP colloids were examined in the presence of three bacterial species: *S. aureus* (Gram-positive), *P. aeruginosa* (Gram-negative) and *E. coli* (Gram-negative). Visual and spectroscopic analysis of the ATB@AuNP colloids before and after exposure to bacteria was used to determine successful pathogen detection. Previously, absorption of prokaryotic cells has been well documented to lie outside the SPB wavelength range monitored in this work.^41–43^ Ideally, upon binding to the bacteria, a visible colour change will be noted, as well as a variation in the intensity and *λ*_max_ of the AuNP SPB. This colour change can be attributed to the binding of the modified ATB surface to the pathogen. Upon binding between the ATB and the bacterial cell wall, agglomeration of the AuNP will result, affording a characteristic red shift in the SPB absorption (Fig. S10†). These variables should remain unchanged if binding between ATB@AuNP and bacteria is not possible – most commonly if the ATB ligand is not sensitive to a specific bacterial genus. Importantly, the *λ*_max_ of the SPB for all four ATB@AuNP colloids remains unchanged after 30 days of storage, showcasing the high stability of these nanocomposites. Representative UV-vis spectra for cepha@AuNP and peni@AuNP are shown in Fig. S11.†

**Fig. 5:**
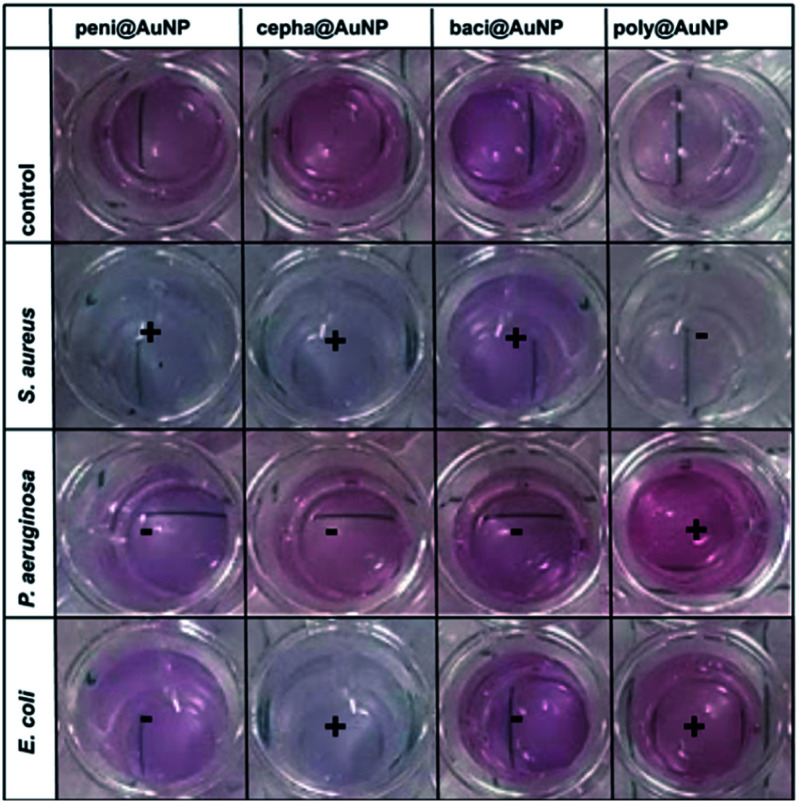
SPB colour changes of ATB@AuNP upon exposure to *S. aureus*, *P. aeruginosa* and *E. coli*, respectively. A positive sign (+) is indicative of a detectable colour change of the ATB@AuNP, binding of bacteria with the conjugated ATB molecule and successful bacterial detection. A negative sign (−) indicated no perceivable colour change and, thus, no bacterial interaction or detection.

**Fig. 6:**
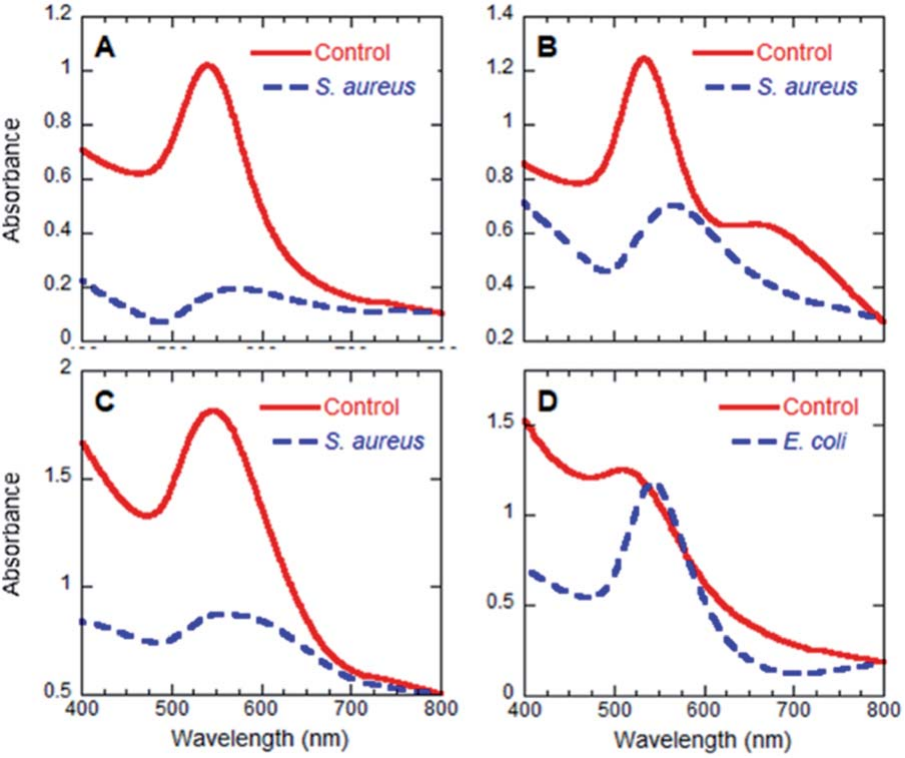
UV-visible spectra illustrating positive detection of bacteria for peni@AuNP (A), cepha@AuNP (B), baci@AuNP (C) and poly@AuNP (D). *S. aureus* is used as an example in A, B and C, while *E. coli* is used in D.

Initial, visual inspection of the colloids following introduction of the three bacterial suspensions clearly showcases the rapid and versatile nature of ATB@AuNP (Fig. 5). The interaction between the bacteria and the ATB@AuNP is expressed as (+) if a colour change is observed, while (−) is reserved for no discernable variation. The anticipated colour changes align with the bacterial sensitivity of the ATB: penicillin (Gram-positive), cephalexin (Gram-positive and some Gram-negative), bacitracin (Gram-positive), polymyxin (Gram-negative). Binding between the bacteria and ATB@AuNP decreases the interparticle distances, as compared to the unbound colloidal solutions. Such an interaction will result in colour changes akin to AuNP agglomeration, producing red-shifts and reduced absorption intensity of the SPB *λ*_max_ (Table 2 and Fig. 6). Given this, each bacterium possesses the capability to facilitate change of the SPB absorption in relation to its affinity for the ATB ligand. While all nanospecies may be capable of the colorimetric detection, the current focus is on the spherical nanoparticle SPB (*λ*_max_ ∼520–560 nm). Responsible for the visible pink hue of the colloids and the most largely affected by bacterial affinities. Though several of the ATB/AuNP are comprised of larger, polyhedron shaped nanospecies, SPB absorption attributed to these do not significantly comprise the UV-visible spectra presented in Fig. 6. The triangular nanoplates, observed in both cepha and baci-modified AuNP, typically present SPB > 600 nm.^44^ Similarly, hexagonal gold nanoplates, seen for peni, cepha and baci@AuNP, commonly present as largely red-shifted SPB (>700 nm).^45^ While a SPB at 690 nm is observed for baci/AuNP, likely attributed to the presence of the triangular and hexagonal nanoplates, no variation in this band is observed upon bacterial exposure, while variation in the 557 nm SPB is drastically changed following bacterial coordination.

Fig. 5 displays several colour variations following introduction of bacteria to the AuNP colloid. In summary, peni@AuNP exhibits a substantial colour change from pink to blue-violet following exposure to *S. aureus*, while no detectable change was observed upon exposure to *P. aeruginosa* or *E. coli*. A similar colour change was observed for cepha@AuNP in the presence of *S. aureus* and *E. coli*. This observation is coincident with the known antibacterial activity of penicillin G (benzylpenicillin) and cephalexin, respectively, through attachment to the penicillin-binding proteins in Gram-positive bacteria.^46^

The baci@AuNP responded to *S. aureus*, evidenced by a tangible colour change from pink to violet. This result is most likely ascribed to the undecaprenyl pyrophosphate binding capability of bacitracin (a polypeptide antibiotic) that hinders cell wall biosynthesis,^47^ a feature of some Gram-positive bacteria. On the other hand, SPB variations of poly@AuNP were detected in the presence of *P. aeruginosa and E*. coli. This transformation can be credited to the selective binding of polymyxin to lipopolysaccharides predominantly present in Gram-negative bacteria.^48^ All three bacteria implemented in this work produce differing colorimetric patterns, enabling rapid bacterial detection in the presence of an appropriate ATB@AuNP control (in the absence of bacteria).

UV-visible spectroscopy was also used to confirm bacterial detection. As is shown in Fig. 6, all four of the colorimetric changes perceived by the naked eye were also observed in the corresponding spectroscopic data. A red shift (shift to longer wavelengths) of the *λ*_max_ of the AuNP SPB was detected concomitant with the positive visual responses. This wavelength variation can be attributed to AuNP aggregation. This experimental result further substantiates reduced interparticle distance immediately following binding of the ATB@AuNP and bacteria. Assays employing the four ATB@AuNP using lower bacteria concentrations (10^2^ CFU mL^−1^; Fig. S12†) also afforded a decrease in absorption intensity and red-shifting of the SPB, showcasing the sensitivity and advantage of the described methodology.

To date, few studies have exploited the bacterial detection capability of pharmaceutical-conjugated nanoparticles. The current design focuses on hybrid stability and implementing ATB functional groups that binds to specific sites on the bacterial envelopes. A similar technique has been explored for magnetic nanoparticles, combining nanospecies with fluorescent molecules and vancomycin for the rapid detection low bacteria concentrations.^49^ However, several drawbacks of this method include low sensitivity of the fluorescence spectroscopic analysis and the multiple step synthetic design. A complimentary report ascribes successful bacteria identification, in part, to a visible AuNP colour change (from red to blue).^50^ This colour change occurs upon binding of the H2 receptors of the cysteine@AuNP and the bacterial wall. While a step in the right direction, this technique lacks selectivity given the known aptitude of cysteine to bind to a variety interfering molecules present in reaction media. This shortcoming can be addressed through the current methodology given the specificity of the ATB for specific bacteria. Combined with the well-established stability of the ATB under a number of conditions,^51^ the current method offers several advantages compared to previously published routes. The purpose of this work is to ensure that the ATB protectant is able to effectively interact with bacteria upon exposure. While minor modifications of the ATB surface protectants can be expected, as evidenced by FTIR, the primary binding modes of biological molecules (van der Waals and coulombic)^20^ are not favourable to significant structural variations. Furthermore, the positive bacterial detection suggests no substantial changes to the active portion of the ATB have occurred throughout the synthetic procedure. However, limitations for the proposed method have yet to be fully recognized. Future directions should be focused on the detection of other strains of clinical importance, such as *Mycobacterium* – a bacterium that experiences slow growth *in vitro*. Also, the possibility of false positives due to nanoparticle aggregation in a myriad biological fluids or food liquids should be take into account.

## Conclusions

The current contribution describes the straightforward and efficient synthesis of AuNP functionalized with a series of cost-effective and commercial antibiotics: penicillin, cephalexin, bacitracin and polymyxin. Care was taken to include ATB that encompass common strains of both Gram-positive (*S. aureus*) and Gram-negative (*P. aeruginosa* and *E. coli*) bacteria. The ATB@AuNP synthesis was monitored using a number of experimental techniques. UV-visible spectroscopy clearly showcases effective synthesis of the ATB functionalized AuNP, with surface plasmon bands associated with the nanospecies appearing between 520–550 nm. The size of the gold nanospecies was corroborated through the use of DLS and TEM, while the identity of the metallic nanospecies was determined through a cooperative study using HR-TEM, diffraction patterning and EDS. Closer analysis of the nanostructures revealed the formation of multi-population AuNP colloids, with penicillin-, cephalexin- and bacitracin-coordination facilitating small quantities of larger, polyhedral particles (hexagonal, triangular). However, the major AuNP population constituted small, monodisperse spherical AuNP on the average of 2–5 nm. Polymyxin-doped AuNP afforded a singular particle population, spherical and monodisperse, on the order of 4 nm. Baci@ and poly@AuNP colloids also presented as spherical amorphous structures, a result of antibiotic emulsification, which were found to encapsulate the observed gold nanostructures.

UV-visible data illustrated considerable deviations of the SPB absorption wavelength and intensity, but could also be detected by the naked eye, without an instrument. These observations support the capacity of ATB@AuNP to selectively detect a series of bacterial contaminants using a simple colorimetric technique observable with the naked eye. Given this, development of this technique will continue to be explored, with focus on expansion towards a more portable and higher through-put detection methods (*i.e.*, reaction test strips).

## Author contributions

Manuscript preparation, review, and redaction: M. J. Silvero, M. C. Becerra, L. Graham, G. L. Hallett-Tapley; experimental conception: M. J. Silvero; formal analysis and investigation: M. J. Silvero, C. N. Elliott, L. Graham, J. C. Bennett, G. L. Hallett-Tapley; supervision: G. L. Hallett-Tapley, M. J. Silvero; funding acquisition and encouraging to the research: G. L. Hallett-Tapley. All authors have approved the final version of the manuscript.

## Conflicts of interest

The authors declare no conflict of interest.

## Supplementary Material

RA-011-D1RA01316E-s001
