# Supplementary material for: Facile synthesis of antibiotic-functionalized gold nanoparticles for colorimetric bacterial detection
Source: RSC Adv. 2021 Apr 15;11(23):14161–8. doi: 10.1039/d1ra01316e (PMC8697705; doi:10.1039/d1ra01316e)
Supplement: RA-011-D1RA01316E-s001 [file RA-011-D1RA01316E-s001.pdf]

Electronic Supplementary Material (ESI) for RSC Advances.
This journal is © The Royal Society of Chemistry 2021

FACILE SYNTHESIS OF ANTIBIOTIC-FUNCTIONALIZED GOLD NANOPARTICLES FOR COLORIMETRIC BACTERIAL DETECTION

Charlotte N. Elliott,[a] María Cecilia Becerra,[b,c] J. Craig Bennett,[d] Lori Graham,[e] María Jamzín Silvero,[b,c*] Geniece L. Hallett-Tapley[a*]

[a]*Department of Chemistry, St. Francis Xavier University, P.O. Box 5000, Antigonish, Nova Scotia, Canada*

[b]*Departamento de Ciencias Farmacéuticas, Facultad de Ciencias Químicas, Universidad Nacional de Córdoba, Córdoba, X5000. Argentina*

[c]*Instituto Multidisciplinario de Biología Vegetal, IMBIV, CONICET, Argentina.*

[d]*Department of Physics, Acadia University, P.O. Box 49, Wolfville, Nova Scotia, Canada.*

[e]*Department of Biology, St. Francis Xavier University, P.O. Box 5000, Antigonish, Nova Scotia, Canada.*

**Corresponding Authors: jazmincompagnucci@gmail.com, ghallett@stfx.ca*

Table of Contents:

Figure S1 .... S2
Figure S2 .... S2
Figure S3. .... S3
Figure S4. .... S3
Figure S5. .... S4
Figure S6 .... S5
Figure S7 .... S6
Figure S8 .... S7
Figure S9 .... S8
Figure S10 .... S9
Figure S11 .... S9
Figure S12 .... S10
Figure S13. .... S11

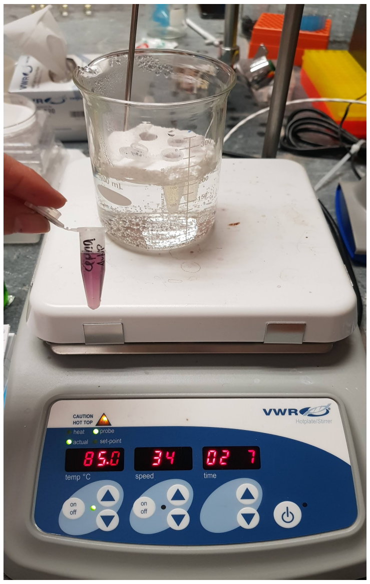

Figure S1. Experimental apparatus equipped with a mild temperature water bath for ATB@AuNP synthesis.

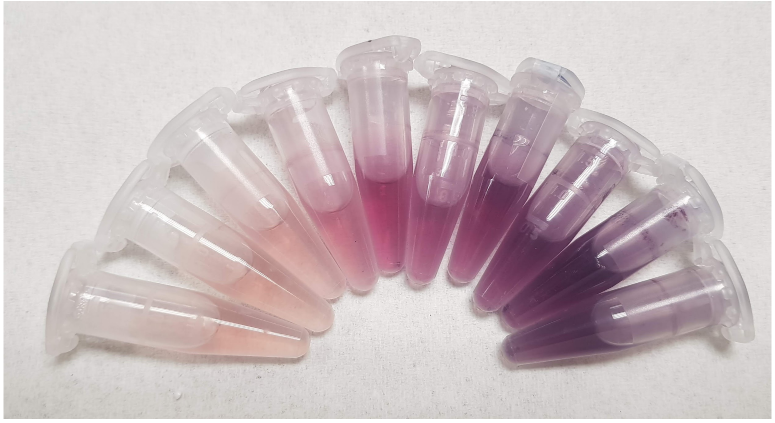

Figure S2. Colloidal solutions of penicillin functionalized AuNP synthesized using various concentrations of penicillin.

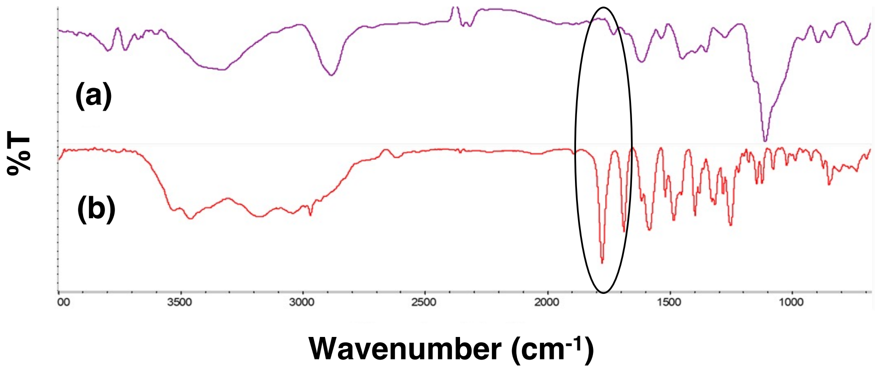

Figure S3. Representative FTIR spectra of (a) cepha@AuNP and (b) cepha ATB only. Note the disappearance of the characteristic amide frequency at 1690 cm^{-1} upon binding to the AuNP surface.

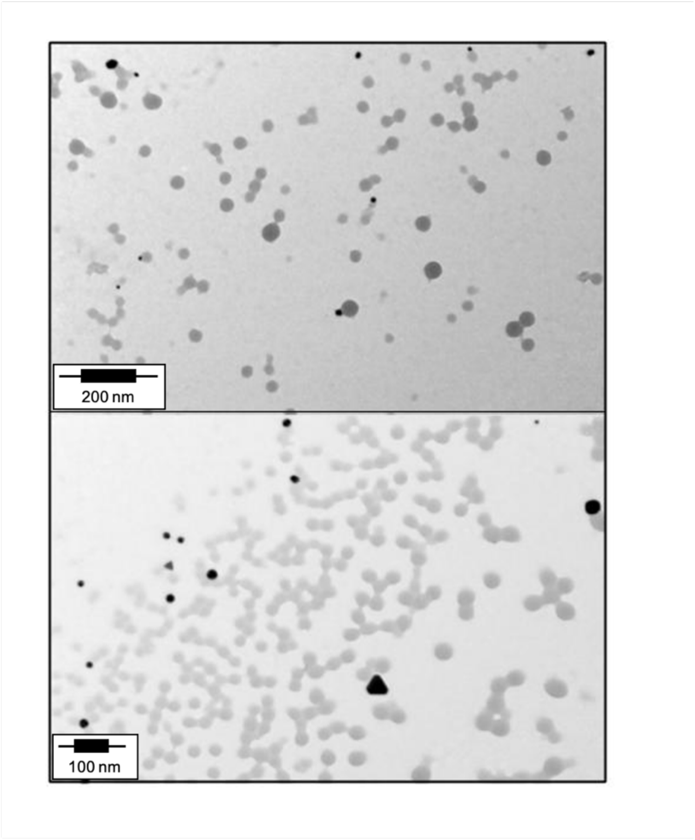

Figure S4. Nanoemulsion formation in baci@AuNP solutions. Note the aggregation of several nanodroplets with each other.

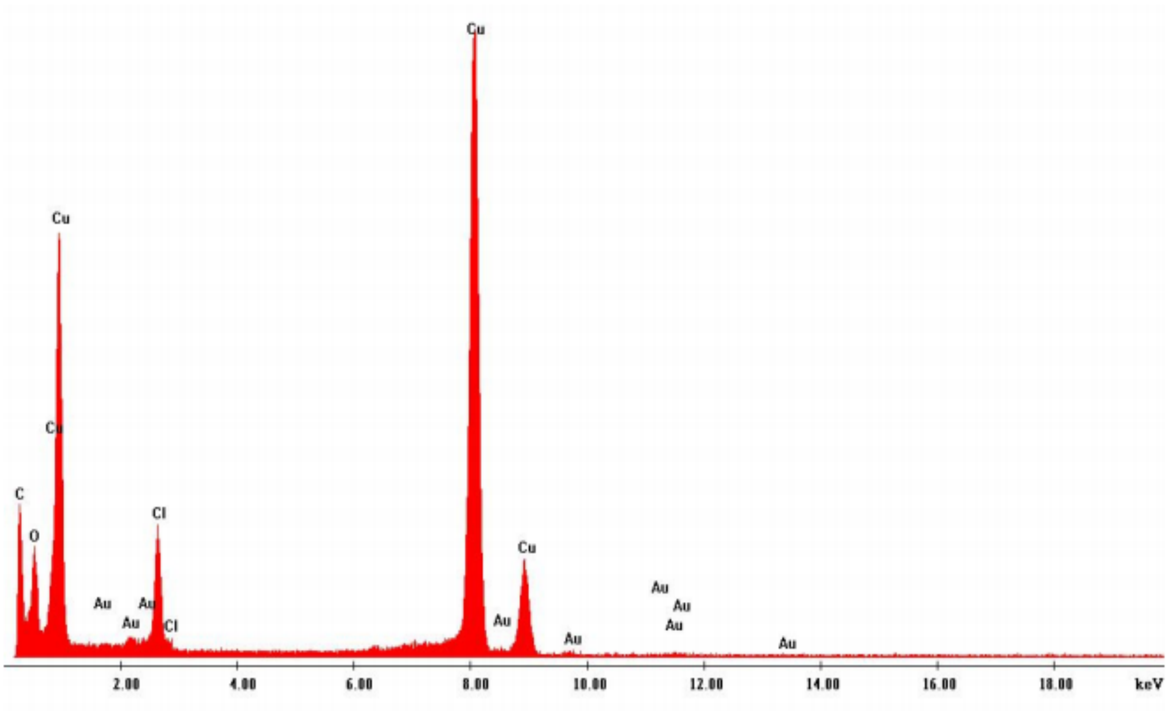

Figure S5. Energy dispersive X-Ray spectrum of the selected area following diffraction pattern analysis. Note the presence of elemental Au supporting identification as a AuNP species.

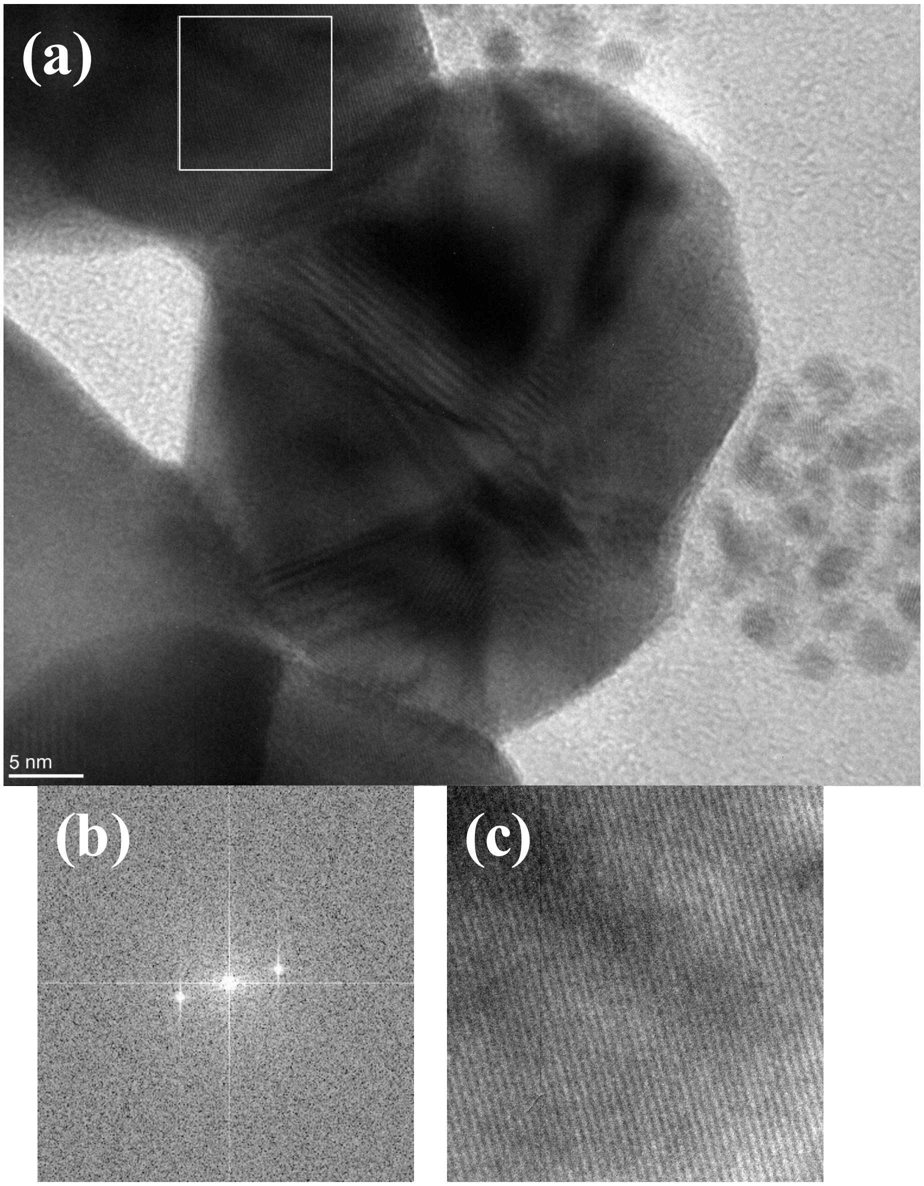

Figure S6. (a) HR-TEM, (b) FFT and (c) filtered inverse FFT for cepha@AuNP. Filtered inverse FFT clearly shows the lattice fringes supporting formation of crystalline Au nanospecies.

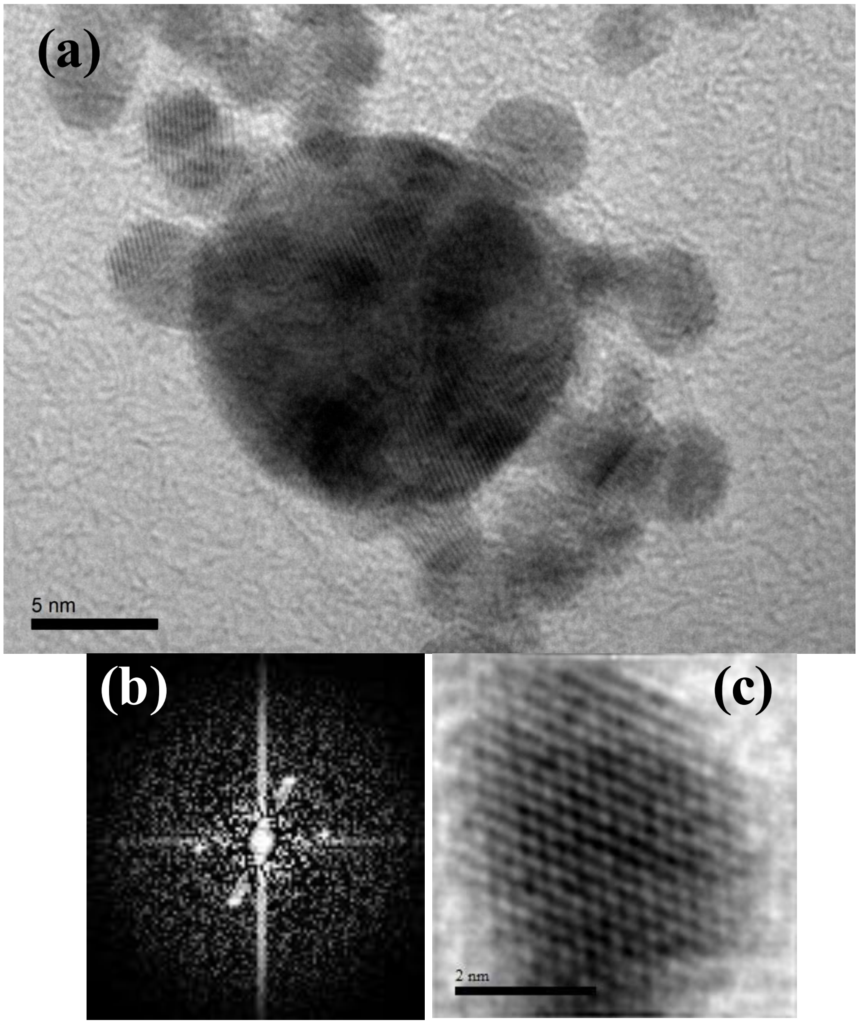

Figure S7. (a) HR-TEM, (b) FFT and (c) filtered inverse FFT for peni@AuNP. Filtered inverse FFT clearly shows the lattice fringes supporting formation of crystalline Au nanospecies.

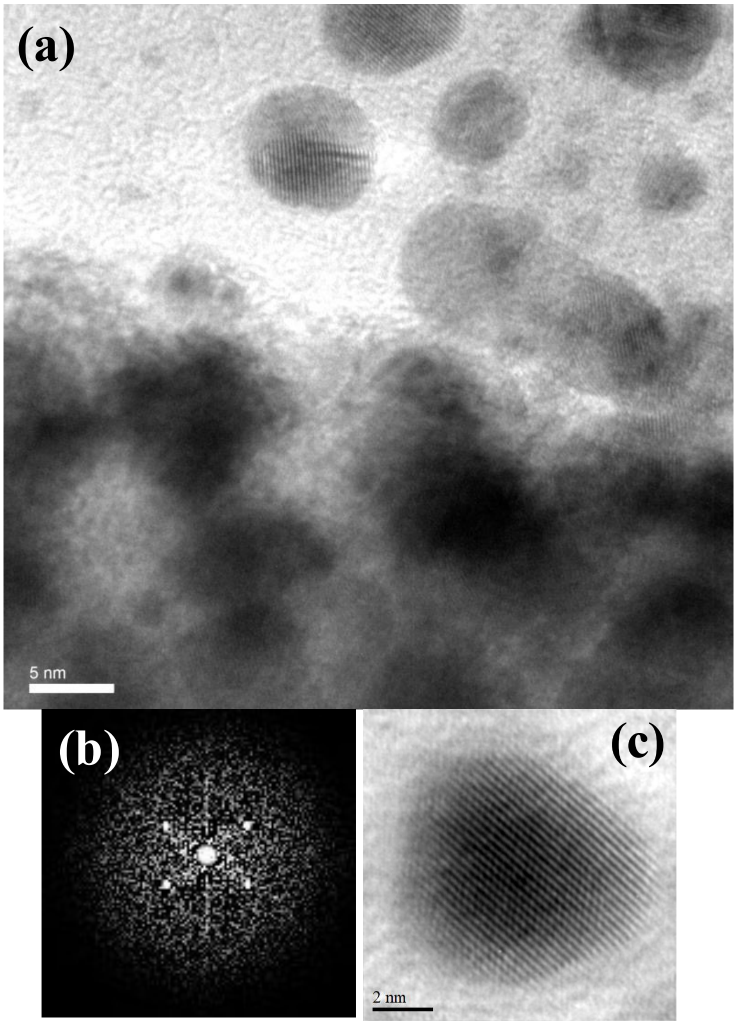

Figure S8. (a) HR-TEM, (b) FFT and (c) filtered inverse FFT for poly@AuNP. Filtered inverse FFT clearly shows the lattice fringes supporting formation of crystalline Au nanospecies.

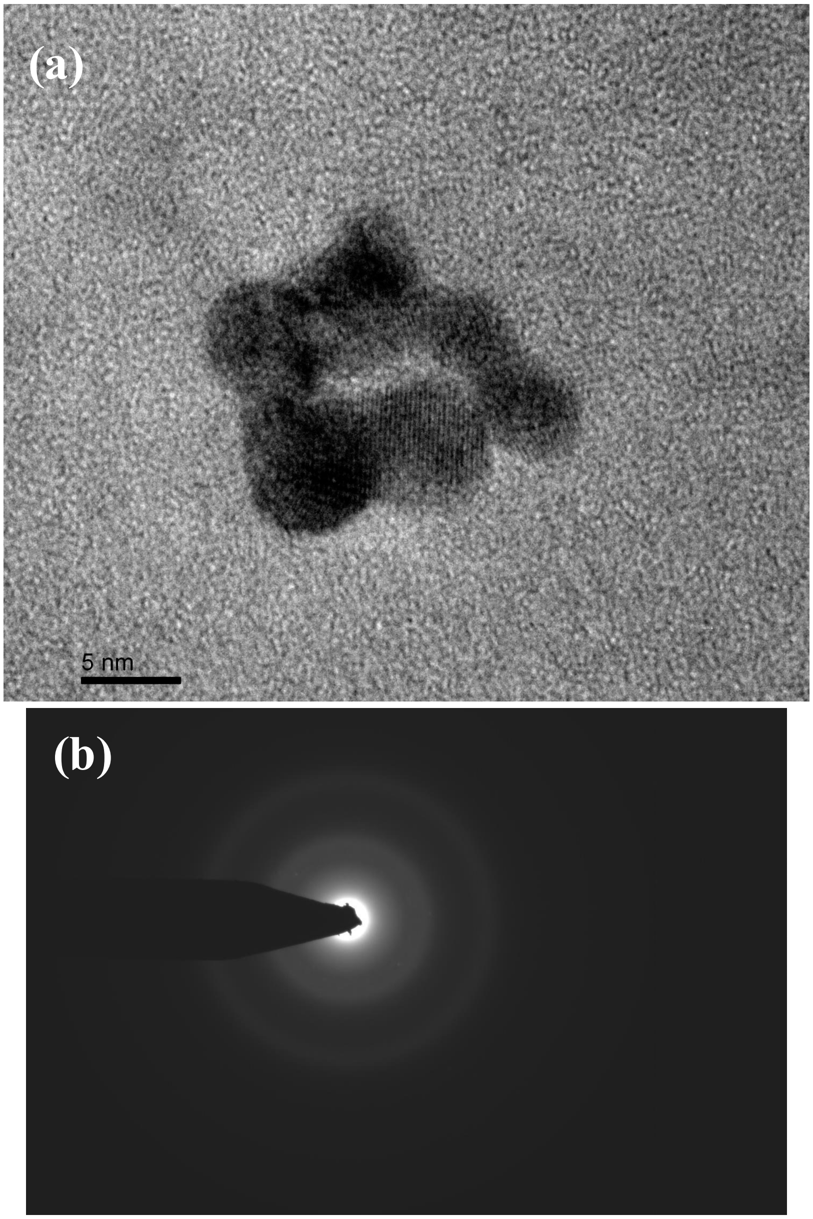

Figure S9. (a) HR-TEM and (b) diffraction ring patterning for baci@AuNP. Diffraction ring analysis confirms the Au nature of the nanospecies with the diffraction rings being representative of the Au (111) surface.

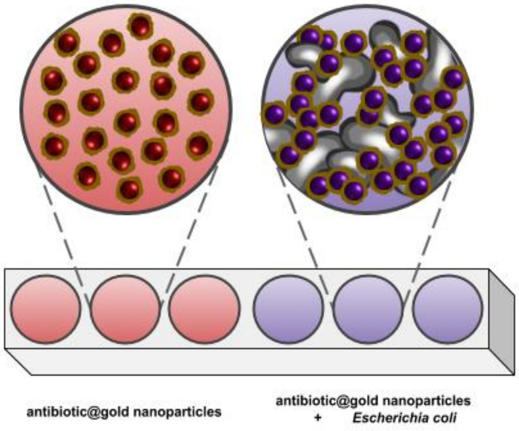

Figure S10. Illustration showcasing the expected mode of colorimetric bacterial detection.

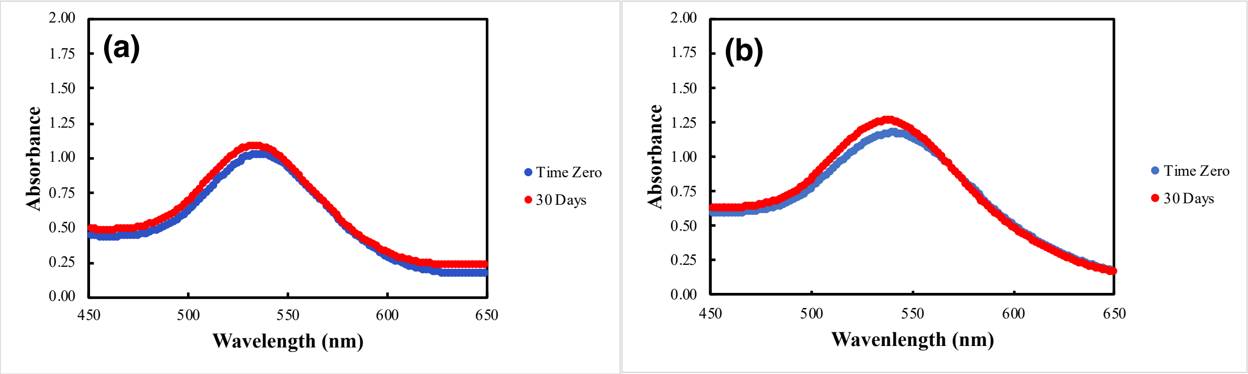

Figure S11. UV-visible spectra of (a) cepha@AuNP and (b) peni@AuNP monitored over a 30-day period to assess stability of the ATB@AuNP colloids.

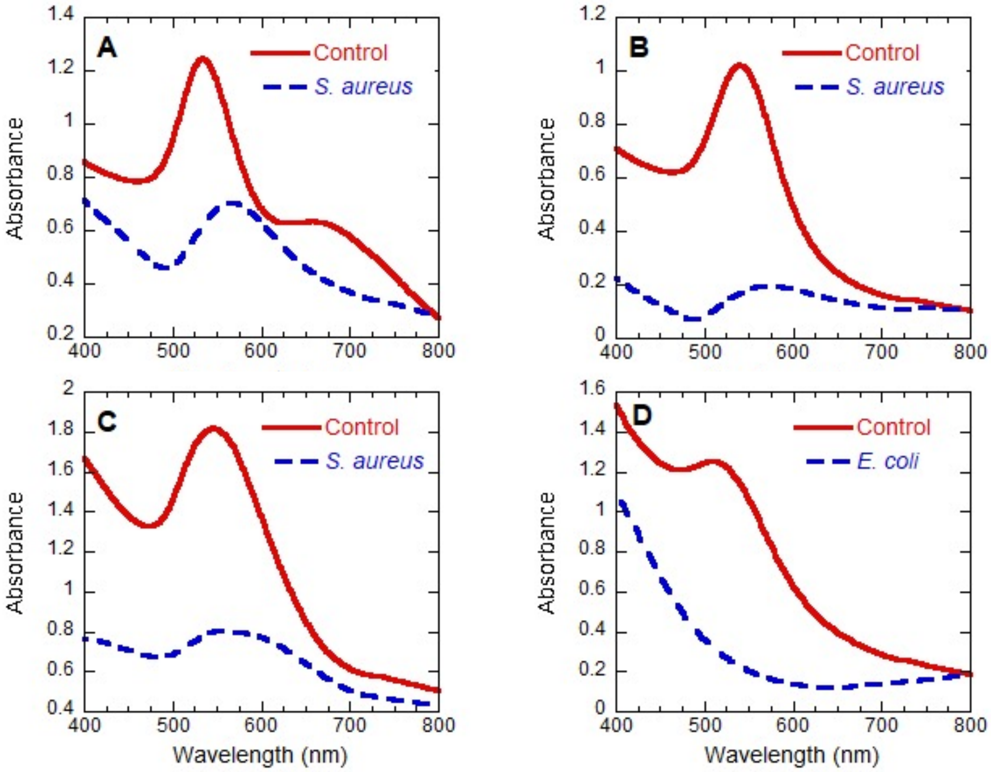

Figure S12. UV-visible spectra of (a) cepha@AuNP, (b) peni@AuNP and (c) baci@AuNP following addition of 10^2 CFU/mL of *S. aureus* and of (d) poly@AuNP following introduction of *E. coli*.

$$\text{mol HAuCl}_4 \bullet 3\text{H}_2\text{O} \times \frac{196.97\frac{\text{g}}{\text{mol}}\text{Au}}{393.83\frac{\text{g}}{\text{mol}}\text{HAuCl}_4 \bullet 3\text{H}_2\text{O}} \quad \textbf{(1)}$$

$$\text{mol } \cancel{\text{HAuCl}_4 \bullet 3\text{H}_2\text{O}} \times 0.5001 \frac{\text{Au}}{\cancel{\text{HAuCl}_4 \bullet 3\text{H}_2\text{O}}}$$

$$\therefore \ \mathbf{mol\ HAuCl_4 \bullet 3H_2O \ \times 0.5001 = mol\ Au}$$

$$r_{AuNP} = {}^{d_{AuNP}}/_{2} \quad \textbf{(2)}$$

$$N_{Au} = \left(\frac{r_{AuNP}}{0.144}\right)^3 \quad \textbf{(3)}$$

$$[\text{AuNP}] = \frac{[\text{Au}]}{N_{Au}} \quad \textbf{(4)}$$

Figure S13. Methodology used for the approximation of [AuNP].

The approximate [AuNP] were calculated using approximations of the number of gold atoms and the TEM imaging data. A brief description of the mathematical process is presented herein. First, the concentration of Au is calculated, in M ([Au]) from the known concentration of Au^{3+} salt. **Eq. 1** presents the equations required to convert mol of $HAuCl_4$ to Au. From here, the average radii (r_{AuNP}; nm) of the AuNP can be calculated using the average AuNP diameter measured from TEM images (d_{AuNP}; nm), **eq. 2**. Next, approximate the number of Au atoms (N_{Au}) comprising the nanoparticle structure (**eq. 3**), where 0.144 nm is the average atomic radius of Au. Finally, using **eq. 4,** estimate the approximate concentration of AuNP ([AuNP]; M) using N_{Au} and the approximate [Au] as calculated from eq. 1.
